# Climatic suitability of the eastern paralysis tick, *Ixodes holocyclus,* and its likely geographic distribution in the year 2050

**DOI:** 10.1038/s41598-021-94793-2

**Published:** 2021-07-28

**Authors:** Ram K. Raghavan, Z. Koestel, R. Ierardi, A. Townsend Peterson, Marlon E. Cobos

**Affiliations:** 1 Department of Veterinary Pathobiology, College of Veterinary Medicine, University of Missouri, Columbia, MO 65211 USA; 2 Department of Public Health, School of Health Professions, University of Missouri, Columbia, MO 65211 USA; 3 Veterinary Medical Diagnostic Laboratory, College of Veterinary Medicine, University of Missouri, Columbia, MO 65211 USA; 4 Department of Ecology and Evolutionary Biology and Biodiversity Institute, University of Kansas, Lawrence, KS 66045 USA

**Keywords:** Computational biology and bioinformatics, Zoology, Ecology, Environmental sciences

## Abstract

The eastern paralysis tick, *Ixodes holocyclus* is one of two ticks that cause potentially fatal tick paralysis in Australia, and yet information on the full extent of its present or potential future spatial distribution is not known. Occurrence data for this tick species collected over the past two decades, and gridded environmental variables at 1 km^2^ resolution representing climate conditions, were used to derive correlative ecological niche models to predict the current and future potential distribution. Several hundreds of candidate models were constructed with varying combinations of model parameters, and the best-fitting model was chosen based on statistical significance, omission rate, and Akaike Information Criterion (AICc). The best-fitting model matches the currently known distribution but also extends through most of the coastal areas in the south, and up to the Kimbolton peninsula in Western Australia in the north. Highly suitable areas are present around south of Perth, extending towards Albany, Western Australia. Most areas in Tasmania, where the species is not currently present, are also highly suitable. Future spatial distribution of this tick in the year 2050 indicates moderate increase in climatic suitability from the present-day prediction but noticeably also moderate to low loss of climatically suitable areas elsewhere.

## Introduction

*Ixodes holocyclus*, the eastern paralysis tick of Australia, is a leading veterinarily and medically significant tick species implicated in the potentially fatal tick paralysis to humans, feline and canine hosts^1, 2^. Tick paralysis is a neuromuscular condition which could lead to death in severe cases. In humans, the disease is more commonly reported among children compared to adults, occurring seasonally and regionally in a predictable fashion^3^. Typical symptoms in patients include change in voice, vomiting, lack of appetite and anisocoria^4^. Although well studied among dogs, tick paralysis has also been noted as a significant concern among cats and other animals such as cattle and sheep^5^. The current geographic distribution of *I. holocyclus* lies along the eastern coast of Australia, from northern Queensland all the way to the Shire of East Gippsland in Victoria (Refer to Fig. 2 of Barker and Barker^6^). These ticks are active throughout the year, with the peak activity of different life-stages overlapping each other, and they are known to parasitize a broad range of wildlife hosts.

Anecdotal reports suggest that incidence of tick paralysis may be increasing in areas where these ticks are known to occur^7^ and the transport of infested pet animals to areas outside the endemic region is a concern^8^. The increase in cases among cats and dogs around urban areas of southeastern Queensland^7^ could be indicative of a few factors at play, including a possible upsurge in the abundance of *I. holocyclus* ticks in its native range resulting in higher contact rates of these ticks with incidental hosts, and possibly also an expansion or shift in their traditional range. Current understanding of the distribution of *I. holocyclus* is based on historic as well as recent acarological surveys^6^. However, the full extent of this species’ spatial distribution may differ from the traditionally known boundaries since geographically limited, periodic field surveys alone cannot determine a species’ distribution in complete detail. Furthermore, ongoing climate change in recent decades could be altering the distribution of this species, as is the case with the closely related *I. ricinus* ticks in Europe^9, 10^ and *I. scapularis* in N. America^11, 12^. For a veterinarily and medically significant vector species such as *I. holocyclus*, it is of immense value to know the areas that are likely currently occupied by this species, as well as those areas that are potentially suitable for future invasion, and possibly establishment, even if the species is currently not observed.

Current and future distribution of a species can be determined using ecological niche modeling approaches, a topic that has been adequately described in the literature in general^13^ and specifically for disease-related systems^14^. In recent years, several studies have used presence-only species occurrence data and environmental data to estimate current distributions of vector species, including ticks^11, 12, 15–18^, and the future distribution of ticks under different climate change scenarios^10, 15–17^. The purpose of this study was to apply these techniques to evaluate the potential current and future distribution under future climate change conditions of *I. holocyclus* in Australia.

## Results

**Figure 1:**
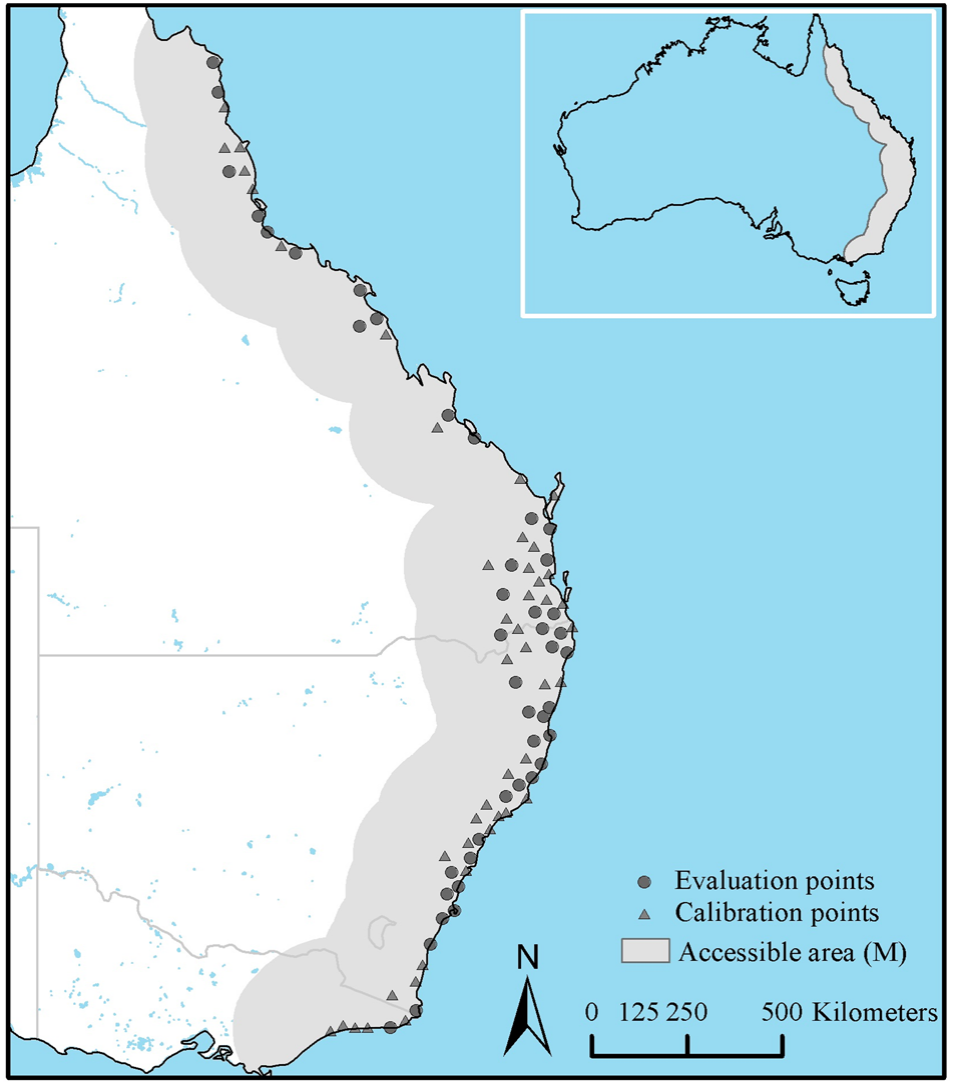
Rarefied occurrence data used for niche model calibration and evaluation, and the areas accessible (**M**) to *Ixodes holocyclus* through natural dispersal.

Of the 2714 records representing *I. holocyclus* occurrences, one record did not include latitude coordinates. The remaining 2713 occurrences were distributed in a north–south pattern narrowly along the east coast of Australia starting near Starcke, Queensland in the north to Bairnsdale/Metung, Victoria in the south. Most occurrences were found near the coast, which are also areas with relatively high human density (Fig. 1). Occurrences were within a median distance of 19.41 km from the coast, and the farthest location from the coast where *I. holocyclus* was positively identified was 274.8 km inland, near Amiens, Queensland. Of the 2713 records, 1827 were representative of ticks collected in the same locality; meaning more than one *I. holocyclus* specimen was collected, and the removal of repeated coordinates resulted in a total of 886 unique geographic coordinates for modeling. Further, the rarefication of occurrences using a 50 km distance between neighboring points left us with a total of 89 unique locations, and no clusters were noticeable in the rarefied data when plotted on a map.

Climate variables selected with the jackknife procedure and the different sets of environmental variables used for constructing niche models are present in Supplementary File 1. In all, 1479 candidate models were built with differing combinations of regularization multipliers, response types (linear, product etc.), and the three environmental variable sets. All the models were significant based on the partial ROC test (*P* < 0.05), of which 6 met the omission rate criterion (< 5% omission of evaluation occurrences). One of the 6 models met both the omission rate and AICc criteria, considered as the final model. This model had a regularization multiplier value of 8, response type set to ‘threshold’, and environmental variable set 1, which included annual precipitation, maximum temperature of the warmest month, temperature seasonality, temperature annual range, annual mean temperature and mean diurnal range. The final model had an omission rate of 0.044% and the AICc value for this model was 1283.5.

**Figure 2:**
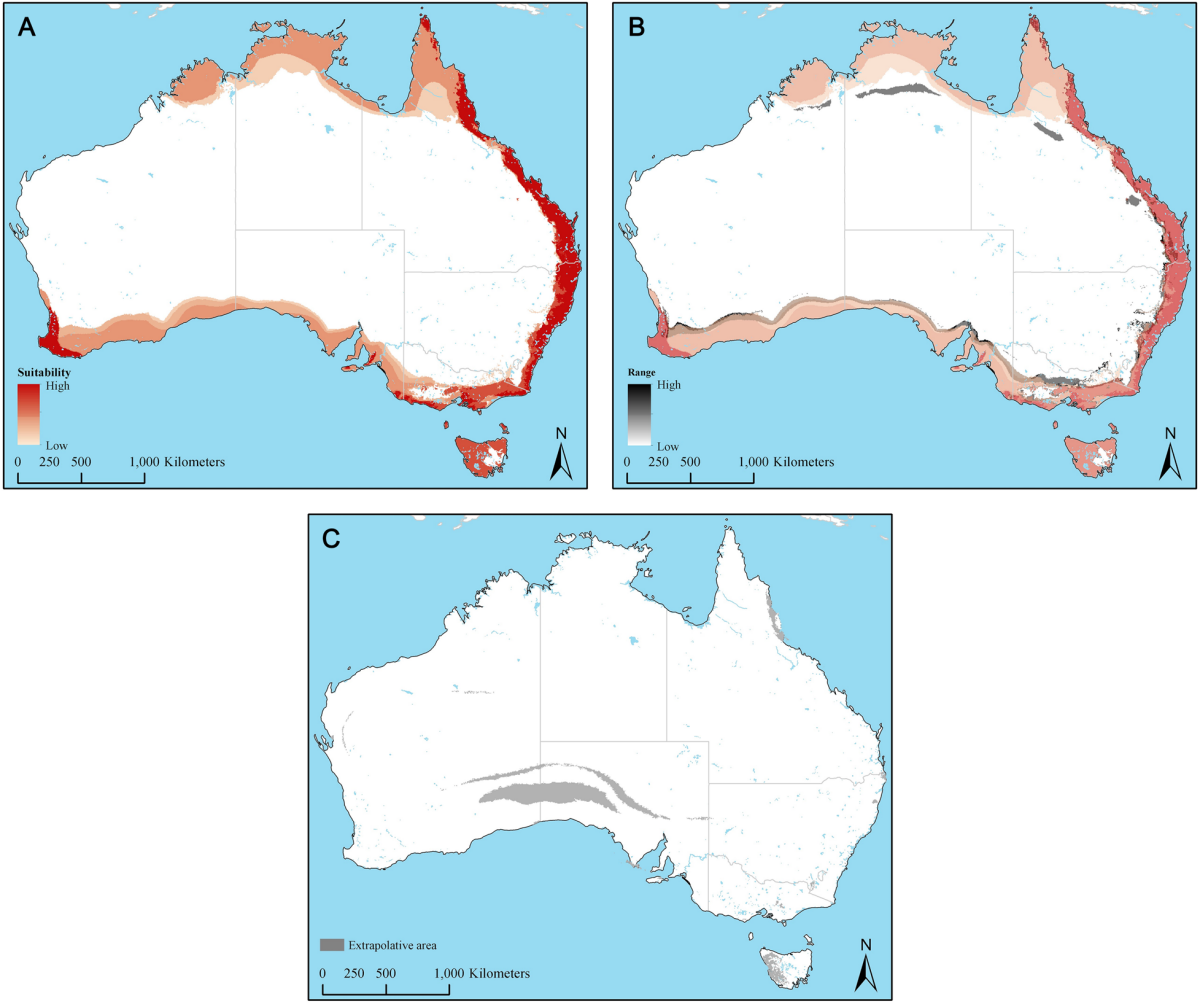
(**A**) Predicted median suitability for *Ixodes holocyclus* under present-day climate conditions. (**B**) Variability associated with predicted suitability under present day climate conditions represented by the range of predictions. Shades of red represent suitability; warmer colors indicate higher values of suitability. (**C**) Mobility-oriented parity (MOP) results under present day conditions, revealing strictly extrapolative areas.

The median prediction for spatial distribution of *I. holocyclus* in Australia based on the replicated final model with bootstrap function is present in Fig. 2A. The model’s uncertainty was estimated using the range, which did not indicate serious concerns, with relatively few areas indicating moderate differences between predicted values (Fig. 2B). The mobility-oriented parity (MOP) analysis revealed areas for which predictions were made purely based on extrapolation (Fig. 2C), which indicated satisfactory model performance with only small swaths of areas outside the predicted presence for *I. holocyclus* in areas of risk of strict extrapolation.

Annual precipitation, (34.2%), maximum temperature of the warmest month (26.23%), and temperature seasonality (16.2%) together explained over 75% of the variability in *I. holocyclus* distribution data in the final model. The remaining environmental variables in set 1, temperature annual range (12.5%), annual mean temperature (6.1%) and mean diurnal range (4.00%) were minor contributors to the final model.

**Figure 3:**
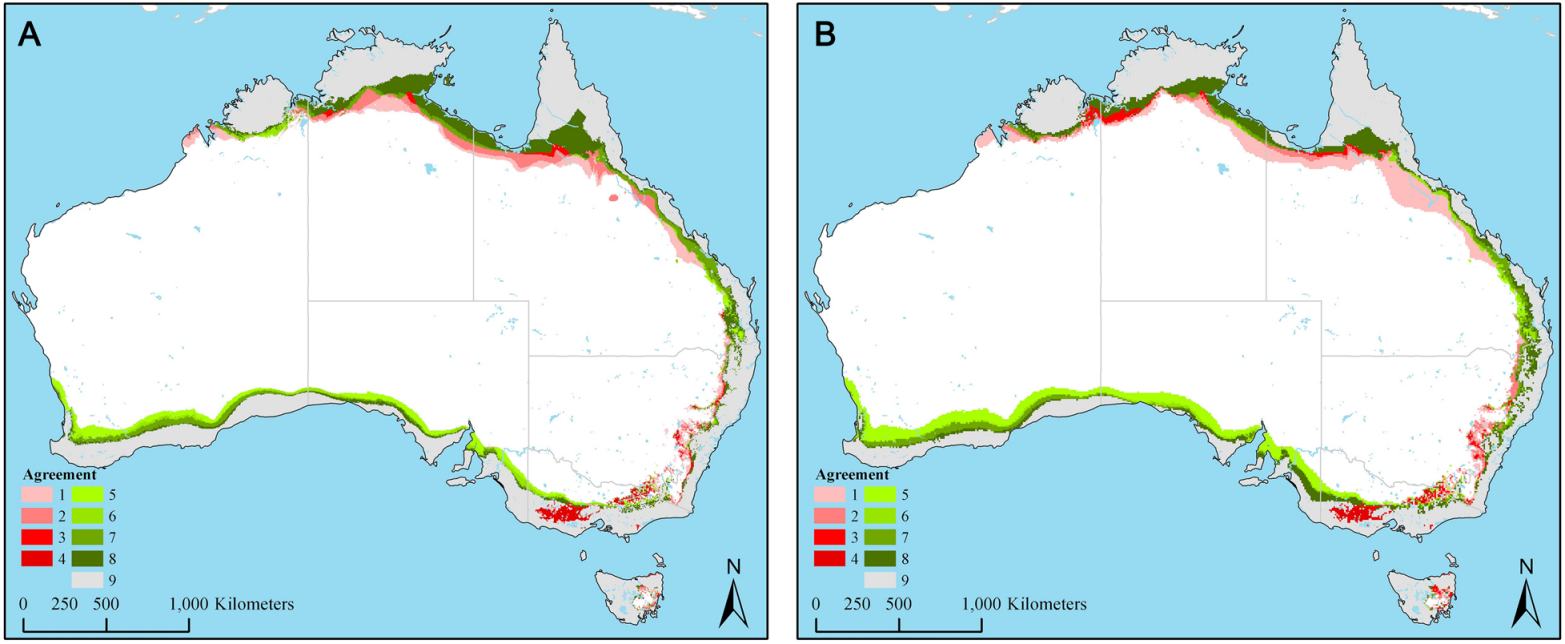
(**A**) Agreement among the general circulation models (GCMs) in predicted future distribution of *Ixodes holocyclus* under the low-emissions representative concentration pathway (RCP) 4.5 scenario. Gray indicates areas under stable suitable conditions. Lighter to darker shades of red indicate areas in which one and progressively more GCMs predicted gains in suitability for *I. holocyclus* distribution under RCP 4.5 scenario. Lighter to darker shades of green indicate areas in which one and progressively more GCMs predicted loss of territory for *I. holocyclus* compared to present day distribution. (**B**) Agreement among the general circulation models (GCMs) in predicted future distribution of *Ixodes holocyclus* under the high-emissions representative concentration pathway (RCP) 8.5 scenario. Gray indicates areas under stable suitable conditions. Lighter to darker shades of red indicate areas in which one and progressively more GCMs predicted gains in suitability for *I. holocyclus* distribution under RCP 8.5 scenario. Lighter to darker shades of green indicate areas in which one and progressively more GCMs predicted loss of territory for *I. holocyclus* compared to present day distribution.

**Figure 4:**
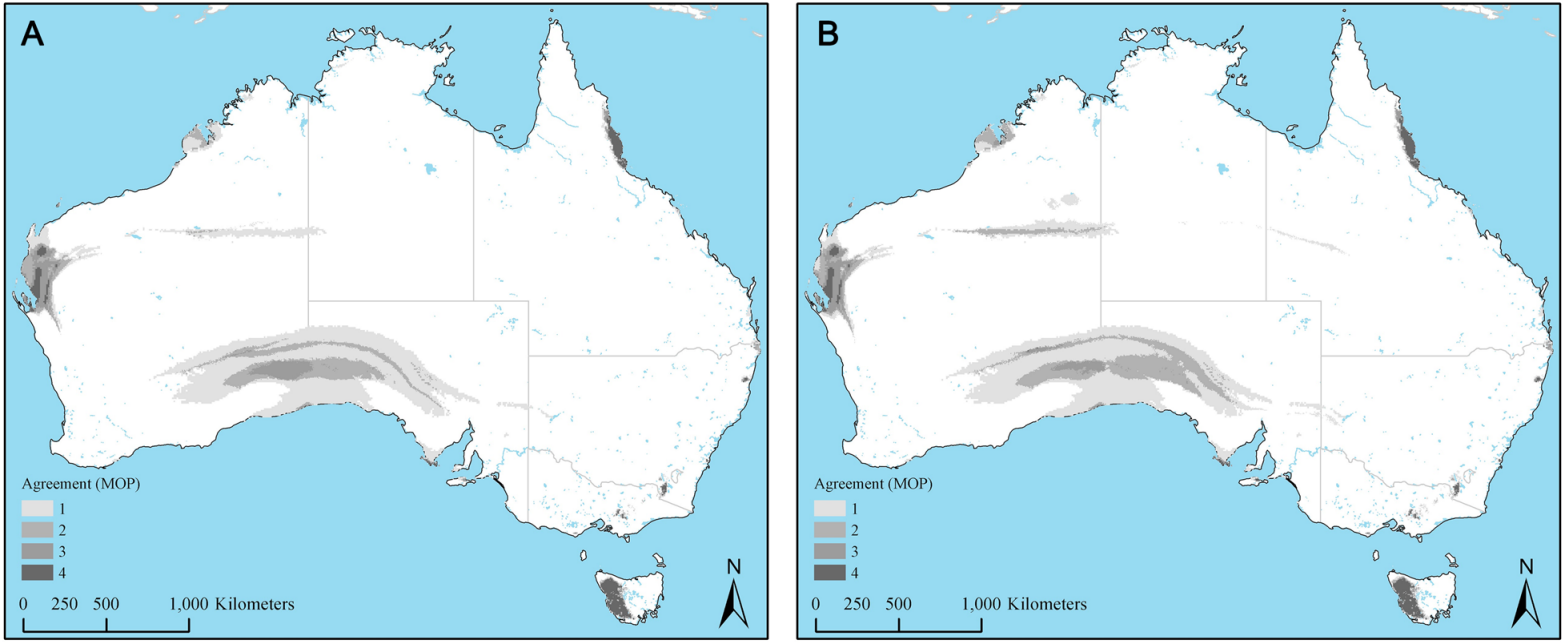
(**A**) Agreement among the four general circulation models (GCMs) in strictly extrapolative areas under future climate change low-emissions scenario (RCP 4.5). Lighter to darker shades of gray indicate the increasing level of agreement of strictly extrapolative areas for *I. holocyclus* derived from the GCMs. (**B**) Agreement among the four general circulation models (GCMs) in strictly extrapolative areas under future climate change high-emissions scenario (RCP 8.5). Lighter to darker shades of gray indicate the increasing level of agreement of strictly extrapolative areas for *I. holocyclus* derived from the GCMs.

The final model, optimized for the present-day conditions that was transferred to the future modeled conditions under the low-emissions, RCP 4.5 and high-emissions RCP 8.5 scenarios are present in Fig. 3A,B, respectively. The corresponding MOP analysis revealing strictly extrapolative areas in the future predictions based on RCP 4.5 and RCP 8.5 scenarios are present in Fig. 4A,B, showing extrapolations occurring mostly in areas that are not suitable for future distribution of *I. holocyclus*.

## Discussion

Tick paralysis is a common tick-borne illness in humans and animals throughout the world, caused by neurotoxins produced in the salivary glands of ticks and secreted into a host during the course of feeding by females and immature stages^19^. Fifty-nine ixodid and fourteen argasid ticks are currently believed to be involved in the transmission of tick paralysis worldwide^19, 20^. In Australia, *I. holocyclus* is considered to be the leading tick species implicated in the transmission of tick paralysis primarily in dogs, but also other species, viz. cats, sheep, cattle, goats, swine and horses. Humans are also occasionally affected, and the disease can be fatal^2, 21^. A second tick species, *I. cornuatus* has also been implicated in the transmission of tick paralysis in Australia; however, it is also considered a minor player in this disease^22^. Given the differences in their biology, distribution, and natural history of these two species, we focused on estimating the spatial distribution of *I. holocyclus* in the present study. We recognize, however, that it is important to consider the distributions of both species for proper epidemiological planning and management of tick paralysis in Australia.

Ecological niche modeling is a well-tested approach for estimating species distributions based on abiotic factors^13, 23^. Several new recommendations have been made in recent years for proper construction of niche models; such as the appropriate thinning of occurrence data^24^, consideration of an accessible area for a species being studied (**M**)^25^, thorough exploration of model complexity^26, 27^, and use of multiple statistical criteria for model selection^28, 29^. We carefully considered all these recommendations to produce a robust spatial distribution model for *I. holocyclus*. The resulting replicated models were fairly consistent in predicting suitability for *I. holocyclus*, as indicated by moderate range estimates (Fig. 2B). Further, the MOP analysis indicated satisfactory performance of the present-day model with extrapolation only in small areas outside the predicted suitable areas. These qualities, along with the model’s very low omission rate (0.044%) gives high confidence in the predicted suitable area for this species in Australia. It will be essential, however, to confirm the actual presence of *I. holocyclus* outside the traditionally known areas through acarological surveys to assess our findings.

The present-day spatial distribution predicted in this study (Fig. 2A) indicates that the geographic areas suitable for *I. holocyclus* match the currently known distribution of this species along the eastern seaboard, but the suitability also extends through most of the coastal areas in the south, and up to the Kimbolton Peninsula in Western Australia in the north. Highly suitable areas are present around and south of Perth, extending towards Albany, Western Australia. Most areas in Tasmania are also highly suitable for this species. The current distribution in the Eastern Seaboard may be wider than the traditionally known extents in some areas compared to Roberts^30^. It is likely that *I. holocyclus* will succeed in establishing permanent populations if introduced into areas that are currently free of them along the southern and northern coasts, and along the southwestern coast of Western Australia and Tasmania. Appropriate prevention of tick movement including pet inspections and quarantine will be necessary to avoid introductions.

Future potential distribution of *I. holocyclus* in year 2050 based on both low- and high-emissions scenarios indicate moderate increases in climatic suitability from the present-day prediction (Fig. 4A,B); but noticeably also moderate to low loss of climatically suitable areas in 2050. This loss could be at least partly attributed to potential future temperature and precipitation conditions exceeding suitable ranges for these ticks in these areas, limiting their ability to survive. Predicted loss of suitable areas in future can also be observed to be irregular, and in some areas, particularly along northern Queensland and in Northern Territory, enveloped between stretches of suitable areas. Our use of relatively coarse resolution data (1 km^2^) limits our ability to thoroughly interpret such phenomenon, but this is likely due to variations in the geography in these areas that respond differently to future climate, as well as the potential increase in ocean temperature and subsequent influences on areas along the coast that may render them unsuitable for this species. Despite the noticeable loss in climatically suitable areas, likely no net loss in area will accrue for this species by 2050.

Teo et al.^31^ assessed present and future potential distribution for *I. holocyclus* using both CLIMEX^32, 33^ and a novel, as-yet unpublished “climatic-range” approach to determine the suitability on monthly intervals. CLIMEX allows users to specify different upper and lower thresholds for climatic parameters, some of which were derived for their study from laboratory evaluations of *I. holocyclus*^34^. The present-day distribution reported in that study resembles our results in identification of a relatively narrow area along the East Coast as suitable; however, much of the northern and northeastern areas along the coast, the coasts of South Australia and southwestern Australia, and Tasmania are reported unsuitable. Their future predictions (2050) of the species’ potential distribution were based on two GCMs (CSIRO MK3 and MIROC-H) climate models, were also markedly different from our predictions, anticipating rather dramatic distributional loss for the species. Such model transfers are challenging, with many factors potentially producing inconsistencies^35^. However, the two studies reflect two fundamentally different classes of ecological niche models; CLIMEX is deterministic, whose predictions are largely constrained by user supplied threshold values for model inputs of physiological tolerance limits of a species^33^, whereas Maxent is a machine-learning correlative approach, in which known occurrences of a species is used in conjunction with environmental layers to determine conditions that meet a species’ environmental requirements, and therefore the suitability of geographic spaces. Although the former (CLIMEX) approach is appealing conceptually, scaling environmental dimensions between the micro-scales of physiological measurements and the macro-scales of geography is well-known to present practical and conceptual challenges^36^.

Different ixodid ticks employ different life-history strategies in response to adverse environmental conditions, including behavioral adaptations, active uptake of atmospheric moisture, restriction of water-loss, and tolerance towards extreme temperatures^37^. Precisely which of these mechanisms *I. holocyclus* utilizes, if any at all, for its survival during diverse temperature and humidity conditions is not clearly known, but it is likely to involve multiple mechanisms. In this sense, the threshold values used by Teo et al.^31^, based purely on laboratory observations may have been overly restrictive, leading to a conservative distributional estimate for this species. Further, because relationships between abiotic variables and species’ occurrences are fairly complex and highly dimensional, a physiological thresholding approach wherein values are set independently for different abiotic parameters may not capture species’ relationships with environments adequately. The correlative approaches employed in the present study are data-driven, and as such may capture more of this complexity, with fewer problems of scaling across orders of magnitude of space and time.

In conclusion, ticks are poikilothermic ectoparasites, whose survival, reproduction and other biological functions are regulated by ambient climatic conditions. Although ixodid ticks are known to regulate their body temperatures by moving about their habitat (vegetation), attempts to model their spatial distribution has resulted in models largely based on climate variables. Nevertheless, other factors such as host availability play a significant role in tick distribution, which unfortunately cannot be readily included in correlative ecological niche models largely because such data are rarely available. These suitability predictions, in addition to being entirely based on large-scale climate, also do not reveal the highly likely heterogeneity in abundance or density in different geographic areas within the realized climatically suitable areas. For these reasons, the distribution maps produced in this study must be used with some caution, and perhaps as a guide to target sampling and not as a substitute for thorough acarological surveys.

## Materials and methods

### Occurrence data

Geographic coordinates representing the positive presence of one of the three post-emergence life-stages (larvae, nymph, and adult) of *I. holocyclus* recorded at different locations were preserved in a digital format in Excel 2013 (Microsoft, Redmond WA). This dataset was checked to remove any errors and subsequently georeferenced to the World Equidistant Conic coordinate system in ArcGIS Desktop 10.6 (ESRI, Redlands CA). The georeferenced occurrences were first filtered to keep only one record per geographic location. Clustering of spatial data could cause spatial autocorrelation and lead to producing biased predictive models^16^. Therefore, we randomly eliminated locations within 50 km from each other using the SDM Toolbox (v 2.2c) in ArcGIS. We considered several distances (10, 25, 50, 75 and 100 km), but our choice of the 50 km distance was balanced by the visual inspection of occurrence data at each step for the absence of clusters among rarefied occurrences and the simultaneous retention of enough data to adequately represent climatic and land cover heterogeneity. This “rarefication” resulted in a set of 89 unique geographic coordinates for modeling. One-half of these points were randomly selected for model building and model validation using a random number generator in Microsoft Excel. A few occurrences (n = 8) were found just outside the boundaries of environmental data (described below) and were relocated to the nearest area that had complete environmental data coverage. Presence of any clustering among the rarefied occurrence data was visually verified by plotting the locations on a digital map. Roughly 50% of the rarefied occurrences (n = 45) were kept for model calibration, and the remaining locations (n = 44) were used in the model evaluation. In the final step, the accessible area to this species, **M**^25^ was calculated using a 100 km circular buffer in ArcGIS, and only areas that overlapped land area were clipped.

### Environmental data

The spatial distribution and phenology of ectothermic arthropods such as *I. holocyclus* are largely influenced by ambient climate, and also to varying degrees by other factors such as land cover and host-level factors. For this study, we used bioclimatic variables available from the WorldClim Global Climate Data project (http://www.worldclim.org/). WorldClim version 1.4 data from this source are available in several raster grid formats, each grid cell representing a “bioclimatic” variable at 1 km^2^ spatial resolution on earth’s surface. Values for each grid or pixel represent different bioclimatic conditions that were derived by interpolation of long-term, monthly temperature and precipitation observations from weather stations, and they represent annual trends, seasonality, and extreme or limiting environmental conditions. Detailed description of the methods used for deriving the bioclimatic variables is available^38^. We chose 30'' resolution for environmental data since that closely matched the spatial resolution of occurrence data available for this study. For model calibration, all environmental data were clipped in ArcGIS to the extent of **M**. For simulating the future distribution of *I. holocyclus* under climate change scenarios, we considered two representative concentration pathways (RCPs), one representing a best case, low-emissions scenario (RCP 4.5) and another worst case, high-emissions scenario (RCP 8.5) to account for the uncertainty associated with regulatory influences on the amount of current and future greenhouse gas emissions. For both scenarios, we used model outputs from four global circulation models (GCMs); CSIRO Mk-3 Climate System Model, the Model for Interdisciplinary Research on Climate (MIROC), Community Climate System Model (CCSM) of the National Center for Atmospheric Research (NCAR), and the Canadian Centre for Climate Modeling and Analysis (CCCMA). These datasets were downloaded from the Climate Change, Agriculture and Food Security—Climate Data Portal (http://www.ccafs-climate.org/) at 30'' resolution.

### Niche modeling

We employed the maximum entropy modeling approach described by Philips et al. (2006) and models were built using the MaxEnt 3.4 software^39^ with the rarefied, presence-only occurrence data and bioclimatic layers. First, we sought to reduce the number of environmental layers using the jackknife procedure in Maxent. We started by building a global model with all of the variables except variables known to have spatial artifacts^40^. An individual variable’s contribution with and without its presence to the full model were noted using the jackknife plot, and the models were refit without one or more of the least contributing variables in sequential steps. Variables retained in the last three jackknife steps were kept as three sets of environmental variables for model calibration.

The exploration of the complexity of variable relationships with the occurrence data and different model constraints, specifically the regularization multiplier and model response types is recommended^26^ using the kuenm R package^41^. We built several candidate models by changing the environmental variable set; the input values for regularization multiplier, which determine how closely the model fits the observations in the environmental space; and response type (linear, product, quadratic, etc.), that determine the type of mathematical function applied in the model^23^. Once all models were built, we used three statistical criteria to select our final model(s). First, all non-significant models were excluded using a partial ROC criterion (*P* > 0.05). Of all the significant models, we ranked models based on the omission rate, and any model with a 10% or higher omission rate was excluded. In the third and final step, all significant models with < 10% omission rate were ranked based on AICc values. All models within 2 AIC units of the minimum value were ranked equally. The selected final model was replicated 10 times using the bootstrap function, and the median output was used for making interpretations.

The final model(s) optimized for present-day conditions was then transferred to future modeled conditions under two climate change scenarios in year 2050, RCP 4.5 and RCP 8.5 with the four GCMs. Here, we assume that the niche relationships will remain the same under the future, changed climate. The mobility-oriented parity (MOP) metric^42^, which reveals areas for which predictions were made based purely on extrapolation, was calculated as an additional measure of uncertainty. The median predicted output obtained through 10 replications for each of these GCMs was used for interpretation under both scenarios, and MOP estimates were determined to identify extrapolative areas. Given the multiple GCMs used, we identified the degree of agreement of models and MOP outputs to represent variability. All of these later were performed using the kuenm R package^41^.

All maps presented in Figs. 1, 2, 3, 4 were generated with ESRI ArcMap 10.8.1 (https://www.esri.com/en-us/home).

## Supplementary Information

Supplementary File 1
