# Supplementary material for: Climatic suitability of the eastern paralysis tick, Ixodes holocyclus, and its likely geographic distribution in the year 2050
Source: Sci Rep. 2021 Jul 28;11:15330. doi: 10.1038/s41598-021-94793-2 (PMC8319185; doi:10.1038/s41598-021-94793-2)
Supplement: Supplementary file 1 [file 41598_2021_94793_MOESM1_ESM.docx]

**Supplementary File 1.**

The jackknife procedure with a global model revealed that bioclim variables 3 (isothermality), 15 (precipitation seasonality), and 17 (precipitation of driest quarter) were the least contributors based on overall model fitness with and without these variables included; and, variables 10 (mean temperature of warmest quarter), and 11 (mean temperature of coldest quarter), were highly correlated with bioclim variable 1 (annual mean temperature). Therefore, these variables were removed and the model refit. This step revealed that variables 6 (minimum temperature of coldest month), 13 (precipitation of wettest month), 14 (precipitation of driest month), and 16 (precipitation of wettest quarter) were the least contributors, which were removed, and a third model was fit. The last two steps revealed variable 4 (temperature seasonality) and 7 (temperature annual range), contributed least and to the overall performance of the model and were therefore removed. Variables kept in the three environmental sets are shown in Table 1.

**Table 1. Environmental variable sets used for niche model calibration.**

| Environmental variable set | Bioclim variables |
| --- | --- |
| Set 1 | Annual mean temperature (bioclim 1), mean diurnal range (bioclim 2), temperature seasonality (bioclim 4), maximum temperature of the warmest month (bioclim 5), temperature annual range (bioclim 7), annual precipitation (bioclim 12) |
| Set 2 | Annual mean temperature (bioclim 1), mean diurnal range (bioclim 2), maximum temperature of the warmest month (bioclim 5), temperature annual range (bioclim 7), annual precipitation (bioclim 12) |
| Set 3 | Annual mean temperature (bioclim 1), mean diurnal range (bioclim 2), maximum temperature of the warmest month (bioclim 5), annual precipitation (bioclim 12) |
